# Recurrence of Bilateral Pleural Effusions in a Case of Yellow Nail Syndrome 12 Years After Surgical Pleurodesis

**DOI:** 10.7759/cureus.107256

**Published:** 2026-04-17

**Authors:** Victor Del Rio, Adrian Michael Lorenzana, Keerthana Keshava

**Affiliations:** 1 Internal Medicine, NewYork-Presbyterian Brooklyn Methodist Hospital, New York, USA; 2 Pulmonary and Critical Care Medicine, NewYork-Presbyterian Brooklyn Methodist Hospital, New York, USA; 3 Pulmonary Medicine, NewYork-Presbyterian Brooklyn Methodist Hospital, New York, USA

**Keywords:** exudative pleural effusion, interventional pulmonology, large pleural effusion, loculated pleural effusion, pleurodesis, rare cause of pleural effusion, video assisted thoracoscopic surgery, yellow nail syndrome (yns)

## Abstract

Yellow nail syndrome (YNS) is a rare disorder characterized by yellow dystrophic nails, lymphedema, and recurrent pleural effusions (PEs). Given its rarity, and limited understanding, few options exist for the management and follow-up of patients who suffer from PEs. While pleurodesis and decortication are effective in most cases, recurrence is still possible, although usually within weeks to months of initial intervention. We present a case of recurrent loculated PE attributed to YNS over 10 years after initial treatment, requiring repeat video-assisted thoracic surgery (VATS) and pleurodesis.

## Introduction

Yellow nail syndrome (YNS) is a disease diagnosed by the clinical trifecta of lymphedema, pleural effusions, and thickened yellow nails [1-3]. Pleural effusions are described as exudative, lymphopredominant, and appear serosanguinous, although sometimes chilous [1,2]. While their presence alone does not warrant intervention, some patients experience severe symptoms and reduced quality of life necessitating medical management [1,2,4]. Initial treatment can consist of thoracentesis or chest tube placement depending on the degree of effusions and severity of symptoms [3]. Although this is only a temporary measure, and in a large systematic review, only one case was found to be effusion-free for at least four years [3]. In these situations, either chest or video-assisted thoracic surgery (VATS) with pleurodesis is recommended as the next step [2,5]. The majority of these patients achieve complete resolution within the first year [3,5,6]. If effusions are still present after pleurodesis, studies have found that repeating pleurodesis still has the highest rate of success with patients requiring two to three repeat interventions on average [4,5]. Unfortunately, few studies have been published concerning the recurrence of any cause effusions or standardized management of YNS-associated PEs. Additionally, monitoring of effusions years after pleurodesis remains scarce as YNS cases are rare and reports of long-term monitoring are rarer yet [3,7]. This is one such case, as this patient with known YNS required repeat VATS pleurodesis 12 years after initial treatment.

## Case presentation

**Figure 1:**
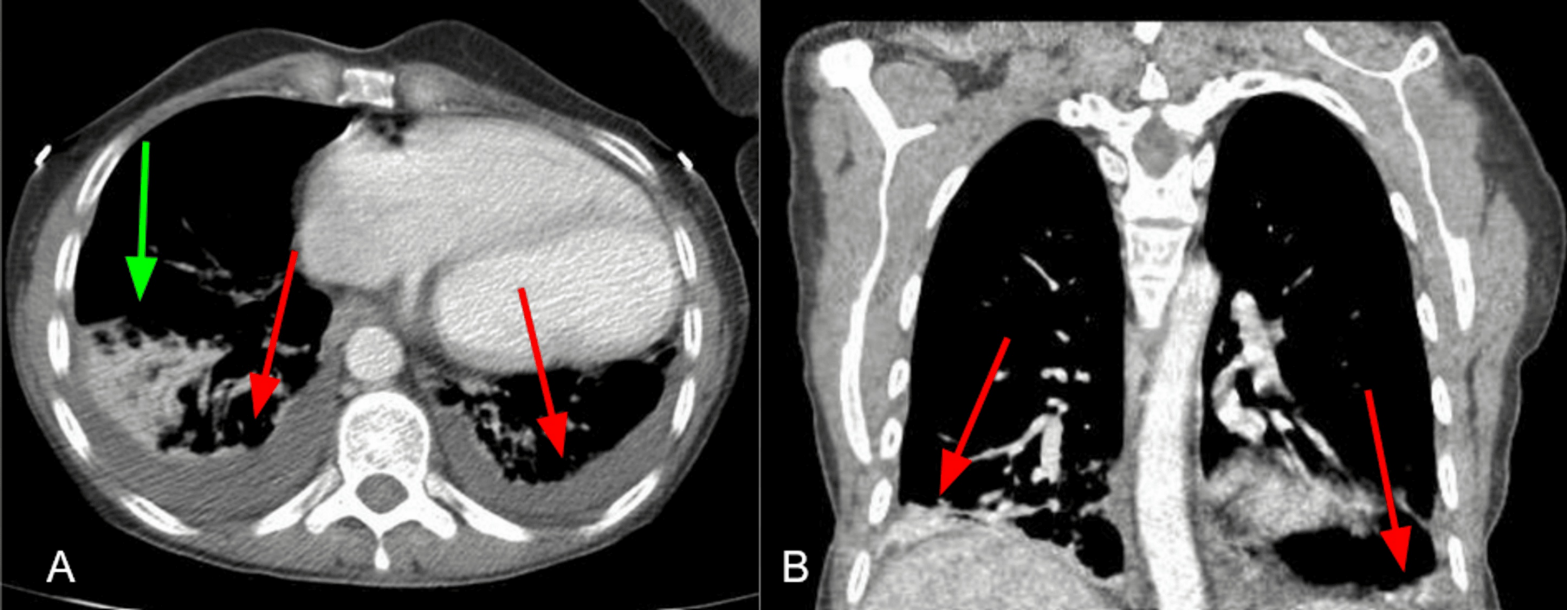
Chest window of CT scan obtained on admission Transverse (A) and coronal (B) view showing small bilateral pleural effusions (red arrows) with compressive atelectasis (green arrow) in A.

A 45-year-old woman with endometriosis, hereditary lymphedema complicated by pericardial effusion (PE) requiring a pericardial window, and bilateral pulmonary effusion requiring bilateral pleurodesis 12 years prior presented to the hospital with worsening left lower quadrant abdominal pain and brown vaginal discharge. She was admitted to the obstetrics and gynecological service for management of stage IV endometriosis. Initial management for vaginal pain and discharge consisted of an infectious workup with a vaginal swab (negative for bacteria or fungi), sexually transmitted infection (*Chlamydia* sp., *Neisseria gonococcus*, *Trichomonas vaginalis* - all negative), and analgesia as the patient did not wish to undergo any surgical interventions for her endometriosis. Her initial CT abdomen and pelvis revealed small bilateral loculated pulmonary effusions worse on the right and dilated loops of bowel with a questionable transitional point concerning for partial small bowel obstruction in the colon (SBO) (Figure 1).

**Figure 2:**
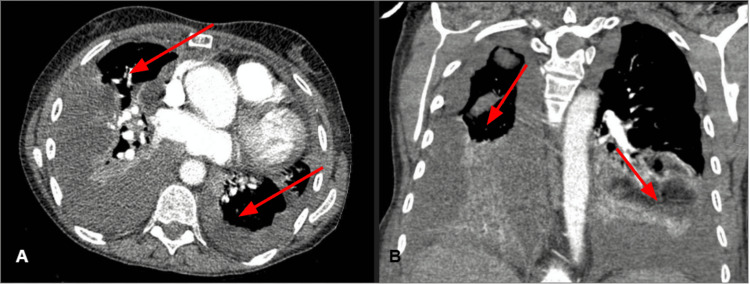
CT scan obtained following abrupt hypoxic episode. Transverse (A) and coronal (B) view of CT chest revealing moderate bilateral pleural effusions (marked by red arrows) CT: computed tomography

By hospital day two, the patient was complaining of progressive abdominal pain worse than prior episodes and now on the left side of her abdomen. On hospital day four, she underwent esophagogastroduodenoscopy (EGD) and colonoscopy after evaluation by the gastroenterology team, which found esophagitis and non-bleeding internal and external hemorrhoids without any colonic obstructions. After endoscopy, she suffered an episode of shortness of breath (SOB) and was found to be hypoxic, requiring oxygen supplementation through the nasal cannula. A CT angiogram scan of the chest was obtained to rule out pulmonary embolism, which revealed loculated small-to-moderate bilateral effusions greater in the right with compressive atelectasis, but no clots (Figure 2).

Initial management consisted of incentive spirometry, titration of analgesia, increased ambulation as tolerated, and diuresis with furosemide. As symptoms did not improve, interventional pulmonology was consulted, who recommended cardiothoracic evaluation for repeat pleurodesis given the recurrence of effusions. While she awaited surgery, a right-sided chest tube was placed on day 10, which drained 700 mL of serosanguinous fluid. Analysis revealed an exudative neutrophil predominant fluid, cytology and cultures were negative for malignancy and infection, as shown in Table 1.

**Table 1:** Pleural fluid studies Pleural fluid obtained after placement of chest tube. Cell differential reveals an exudative serosanguinous fluid with neutrophil predominance. The normal range for cell count was set by the lab agency that collected the samples and tested them. The results range for LDH and total protein is based on Light's criteria, and serum protein is included to establish that this is an exudative effusion based on serum:pleural protein ratio >0.6. RBC: Red blood cell; LDH: lactate dehydrogenase.

Pleural Fluid	Results	Normal range
Color	Red	N/A
Appearance	Cloudy	N/A
RBC count	18,00/uL	0-499u/L
Nucleated cell count	1,950/uL	0-99/uL
Segmented neutrophils	75%	N/A
Lymphocytes	10%	N/A
Mesothelial cellst	Too few to count	N/A
Lactate Dehydrogenase	142 IU/L	<200 IU/L
Triglycerides	98 mg/dL	<50 mg/dL (non chilous effusion) or >110 mg/dL (chilous effusion)
Total Protein	4.3 g/dL	<3 g/dL
Serum protein	5.7 g/dL	

**Figure 3:**
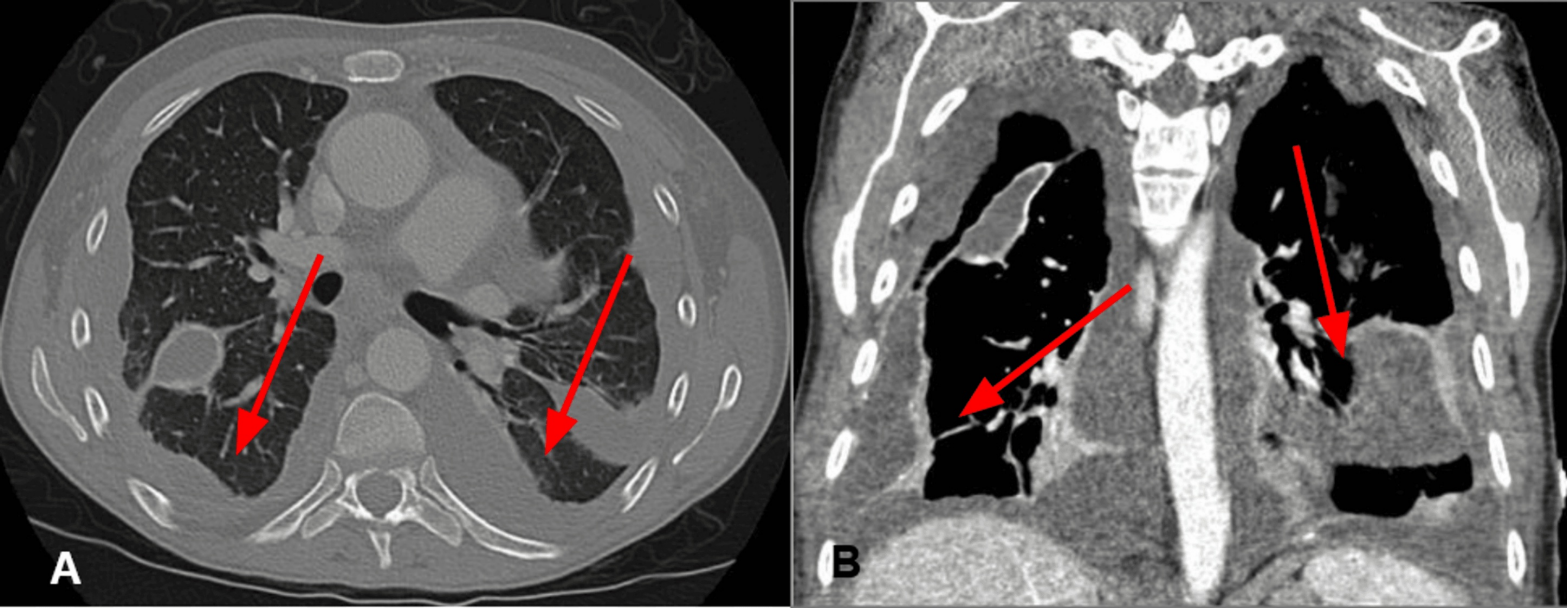
Post VATS CT scan Transverse (A) and coronal (B) view revealing improved and redistributed bilateral pleural effusions (red arrows). VATS: Video-assisted thoracic surgery.

The patient underwent VATS with bilateral pleurodesis, revealing multiple loculated pockets, extensive adhesions from prior pleurodesis, causing lung trapping, and what was described as "chocolate colored studs", which were biopsied. Pathology of these "studs" revealed chronic inflammation with reactive mesothelial hyperplasia without signs of malignancy or endometrial implants. A follow-up CT one week after treatment demonstrated an improvement in bilateral effusions (Figure 3). The chest tube was removed four days after, and the patient was discharged with a stable respiratory status.

Differential diagnosis 

Recurrence of PEs secondary to YNS was the leading diagnosis, given its presence in her medical history. Pulmonary infection was considered, given the respiratory symptoms and effusions, but was excluded by negative blood cultures, sputum cultures, respiratory workup, and pleural fluid cultures. Atelectasis from immobility and splinting due to pain or post-endoscopy complications were suspected, although given the progression and severity of symptoms did not seem likely. Pulmonary embolism had to be ruled out due to the sudden onset of symptoms and was excluded by CT angiogram of the chest. Endometrial implantation into the pleural space was also in the differential list given the patient's known stage 4 endometriosis, presenting complaints, and "chocolate studs" on VATS. This was excluded by biopsy of the pleura and the "studs" during VATS, showing no endometrial implant. Malignant effusion was considered but excluded by negative pleural fluid cytology and pleural biopsy.

Management

Treatment of pulmonary effusions is multimodal and depends on symptom burden and the degree of effusions [1]. In this case, initial SOB and hypoxia occurred after endoscopy and a prolonged hospital stay. As such, diuresis, physical therapy, and incentive spirometry were the initial regimens. If symptoms progress, as with this patient, the next step involves intervention with either thoracentesis or chest tube [1,3]. However, this was a temporary measure in this patient and she required repeat VATS. For complex, loculated effusions, where surgical intervention is not warranted, or wanted by patients, intrapleural fibrinolytic therapy is an adequate alternative [1,3].

Follow-up 

She was followed up one week after discharge at her primary care physician (PCP) and cardiothoracic surgeon's office. At the time, she reported ongoing improvement in functional status, but she was not yet back at her baseline capacity. She denied any further respiratory symptoms and there was no evidence of recurrent effusion. Three months after discharge, she returned to the cardiothoracic surgery office as part of a scheduled follow-up. At this time, she denied any respiratory symptoms, and a chest x-ray obtained revealed clear lungs without effusions.

## Discussion

YNS is characterized by thickened yellow nails, lymphedema, and PEs, although not all three features must be present for diagnosis [3,5]. In this case, the patient had known hereditary lymphedema and recurrent bilateral exudative effusions. Effusions in YNS may be serous or chylous, and diagnostic workup should exclude other pathologies [3]. Maldonado et al. [5] provided a comprehensive review of YNS, analyzing 41 consecutive patients and covering diagnosis and treatment plans for those with PEs. A larger review done by Valdés et al. [3] covered over 150 patients, although this study is limited by the variable quality of case reports and patients without a confirmed diagnosis. It provided valuable insight into the spectrum of disease presentation and variable outcomes based on the chosen management modality. The intensity of treatment is based on the symptom burden. Patients with mild effusions and minimal symptoms may respond to physical therapy and volume control [1]. In more significant cases, chest tube placement or VATS pleurodesis is required [3-5]. Alternative approaches have also been examined; one case report describes the use of a pleurovenous shunt in refractory chylothorax with improvement in both the effusion and lymphedema [6]. In a more novel approach, Matsubayashi et al. examined the use of clarithromycin for the nail changes in YNS with the secondary outcome being the effect on the pulmonary manifestations; while helpful with bronchial symptoms such as cough and sputum production, it did not demonstrate any effect on PEs [8]. This trial also lacked significant power, having only five participants, which prevents generalizability. Regarding recurrence of effusions in YNS, follow-up and management data are limited given the rarity of the condition. Successful resolution occurs in approximately 82% of the patients with YNS and PEs [3]. Vignes and Baran’s review [1] is a relatively recent examination of available literature on YNS, although it does not expand on effusion management and lacks long-term follow-up data. In comparison, a multitude of studies have been published covering interventional approaches to malignant effusions and recurrence rates [9-13]. Established clinical practice guidelines exist for the management and monitoring of malignant PEs, while none are present for YNS [9]. This case demonstrates that recurrence of PEs can happen many years after initial pleurodesis in patients with lymphatic dysfunction.

## Conclusions

YNS is a disease of the lymphatic drainage system and many patients who suffer from it experience pleural effusions. These effusions range in symptom severity and may significantly impact quality of life. For those who experience significant enough effusions, non-invasive treatment is limited and they often undergo chest tube or VATS pleurodesis. In this case, this patient required bilateral VATS 12 years prior to presentation and once again during a new unrelated hospital stay. While VATS has a high effectiveness at permanently treating the effusions, cases of recurrence one year post procedure are rare and less so over a decade. Novel approaches and alternative treatments have been studied, and none show any promising results and most suffer from small sample sizes. This highlights the large gap in information that exists and the need for thorough reviews to create appropriate management and follow-up guidelines.
